# Training LSTM-neural networks on early warning signals of declining cooperation in simulated repeated public good games

**DOI:** 10.1016/j.mex.2020.100920

**Published:** 2020-05-16

**Authors:** Manfred Füllsack, Marie Kapeller, Simon Plakolb, Georg Jäger

**Affiliations:** Institute of Systems Sciences, Innovation and Sustainability Research, University of Graz, Graz, Austria

**Keywords:** Critical transitions, Early warning signals, Long-short-term-memory neural networks, Agent-based model, Repeated public good game, Scale-free networks, Centrality

## Abstract

We present results of attempts to expand and enhance the predictive power of Early Warning Signals (EWS) for Critical Transitions (Scheffer et al. 2009) through the deployment of a Long-Short-Term-Memory (LSTM) Neural Network on agent-based simulations of a Repeated Public Good Game, which due to positive feedbacks on experience and social entrainment transits abruptly from majority cooperation to majority defection and back. Our method extension is inspired by several known deficiencies of EWS and by lacking possibilities to consider micro-level interaction in the so far primarily used simulation methods. We find that

• *The method is applicable to agent-based simulations (as an extension of equation-based methods).*
• *The LSTM yields signals of imminent transitions that can complement statistical indicators of EWS.*
• *The less tensely connected part of an agent population could take a larger role in causing a tipping than the well-connected part.*

## Graphical abstract

**Figure:**
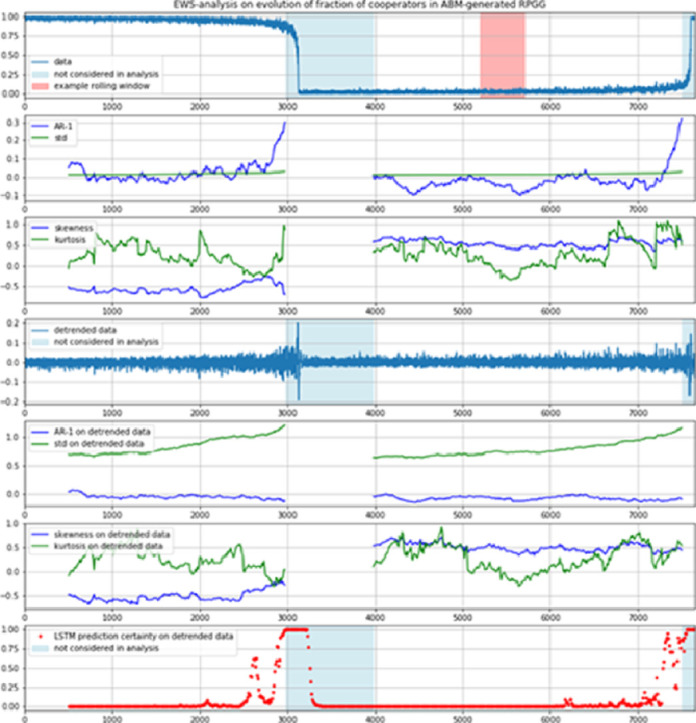

Specifications Table

**Table:** 

Subject Area	Select one of the following subject areas:
*Social Sciences*	*• Social Sciences*
More specific subject area:	*Describe narrower subject area*
	*Systems Sciences*
Method name:	*Please specify a name of the method that you customized.*
	*Early Warning Signals for Critical Transitions*
Name and reference of original method	*If applicable, include full bibliographic details of the main reference(s) describing the original method from which the new method was derived.*
	*Scheffer, Marten, Jordi Bascompte, William A. Brock, Victor Brovkin, Stephen R. Carpenter, Vasilis Dakos, Hermann Held, Egbert H. van Nes, Max Rietkerk, and George Sugihara. 2009. “Early-Warning Signals for Critical Transitions.” Nature 461 (7260): 53–59.*https://doi.org/10.1038/nature08227.
Resource availability	*If applicable, include links to resources necessary to reproduce the method (*e.g. *data, software, hardware, reagent)*

## Introduction

Systems can change behaviour quite abruptly. In the theory of complex dynamical systems sudden regime shifts are grasped as critical transitions (CTs), which occur when gradually drifting parameters transit a threshold value [19]. The rising temperature of water for instance causes abrupt transitions from solid to fluid or to gaseous states at certain temperature values. Theory suggests that such shifts are caused by accumulating positive feedbacks leaving particular traces that can be detected with statistical methods comprised under the term Early Warning Signals (EWS, [5], [6], [31]. These methods, so far, have been applied to real-world- as well as model-generated data. Examples range from ecosystems that show rapid changes in desertification or extinctions of species [32], [34], medical conditions that quickly change from regular to irregular behaviour [26,36], or financial markets that transit from a balanced market to a financial crisis [25]. When considering model-generated data for investigating EWS, most activities so far focus on data from equation-based models (EBMs) [1,^34^,38]. Only rarely, so far, EWS methods have been applied to data from agent-based models (ABMs) [16,18]. This seems at odds when considering the theoretic proposition that CTs are caused by feedback-driven distributed interactions on a system's *micro*-level, which in the aggregated representation at the macro-level as possible with EBMs are only statistically represented, i.e. as an approximation. We claim that certain micro-level details, such as a (possibly) small fraction of free-riders igniting a tipping from cooperation to overall defection in game-theoretic representations of social dilemmas for instance, cannot be detected and comprehended appropriately on the macro-level of EBMs. Investigations into such details of social congestions necessitate the consideration of more fine-grained methods. They demand for simulations with ABMs.

However, since EBMs are directly based on the mathematical concept of differential equation systems, they may be seen closer related to the theoretical foundation of EWSs [19], [20], [21]. Theory in this context seems sound and clear. In practical application however, EWS often remain hidden behind stochastic or parametric particularities of the systems concerned or are not discernible clearly or early enough for waterproof classifications [17]. It seems that if EWS methods should be applicable for predicting CTs in time to avert them, they would need to be significantly refined.

Up to now, such refinement is largely left to human engineering spirit. With the rapid progresses in development and accessibility of automated decision tools however, before all artificial neural networks, hope arises that the predictability of CTs in complex systems can be honed in an at least semi-automated way as well (see also [38]). In order to test the possibility of identifying CT-prone time series with machine learning methods two separate data sets of time series from an ABM of a Repeated Public Good Game (RPGG) were used to assess the predictive power of a Long-Short-Term-Memory Neural Network (LSTM) on previously unseen time series, before all on time series that were generated with RPGGs played in topologically different network settings.

Consecutively, methods applied are explained and the findings are depicted and discussed. The theory of EWS is introduced in Section 2 via a brief outline of stability analysis in dynamical systems. Statistical EWS-methods exploited in the further investigations are detailed, including a discussion of some of the shortcomings related to EWS-detection. Consequently, Section 3 contains a description of the model put to use for the generation of both the training and the test data set. In Section 4 the utilized methods are presented and finally, Section 5 is comprised of a depiction of the results accompanied by a discussion of their significance and furthermore their implications for future research.

## Stability in systems and EWS for critical transitions

**Fig. 1:**
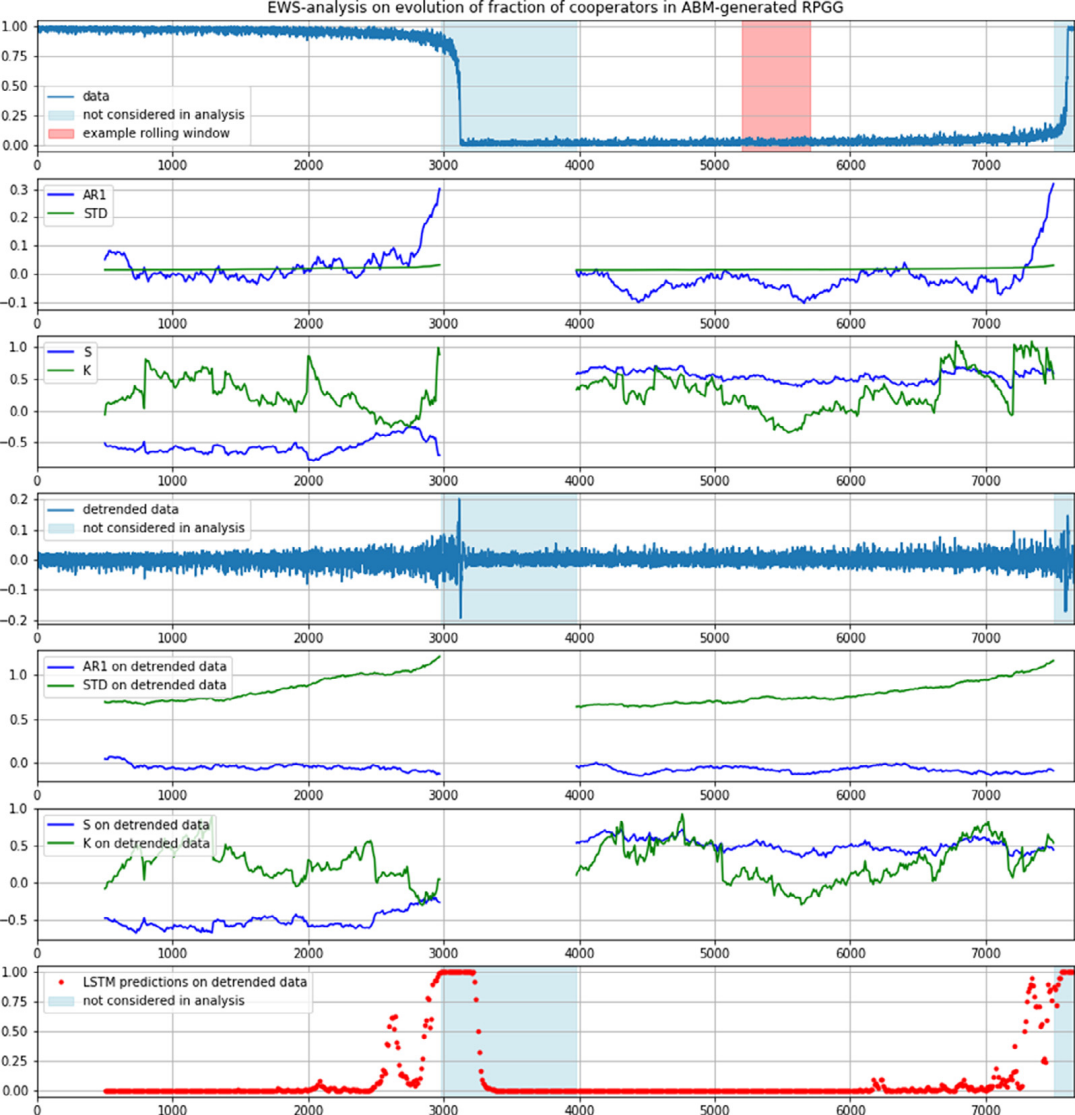
Top plot: example of generated time series showing loss and reinvigoration of cooperation in an RPGG (% of co-operators in the population). Highlighted (in pink) is an exemplary window of 500 steps as extracted in steps of 10 over the whole time series for analysis. Second row plot: autocorrelation at-lag-1 (AR1) and standard deviation (STD) of the un-detrended time series as shown in the top plot, with data immediately before the tipping and shortly afterwards (light blue) not considered to avoid rolling window miscalculations. Third row plot: skewness (S) and kurtosis (K) of the same un-detrended time series. (These EWS-analyses on un-detrended time series are shown for comparison. They are not used in further analyses). Fourth row plot: detrended time series (residuals) of top plot, using a Savitzky-Golay filter with window length 101 and a polynomial order of 5. Fifth and sixth row plots: AR1, STD, S and K of the detrended data. Bottom row plot: LSTM predictions about imminent CT on the detrended time series in the fourth row plot.(For interpretation of the references to color in this figure legend, the reader is referred to the web version of this article.)

Most dynamical systems have one or more so-called equilibria, i.e. stable states. If a system is close to one of those equilibria its eigen-behavior [37] leads to an evolution towards this stable state. This attraction causes systems to return to an equilibrium, even after (small) perturbations [24]. Certain changes in a system can diminish this attraction, so that the return to the stable state takes more time or the system even transitions into an alternative equilibrium. In many systems these changes can be rather abrupt and can lead to sudden regime shifts [19], so-called critical transitions (CTs) (see Fig. 1).

Even though the transition itself is sudden, it is sometimes possible to observe signals that indicate an imminent regime shift. A viable indicator of the approach of such a tipping point is “critical slowing down” (CSD): close to a tipping point, systems are slower to recover from perturbations and return to their original stable state, hinting at a loss of resilience and a possible critical transition [6]. CSD can be seen in different statistical properties of time series. An increase in autocorrelation at-lag-1 (AR1) indicates a higher “short-term memory” of the system, related to changes in the correlation structure close to a tipping point ([7], see 2nd row plot in Fig. 1). In addition to a signal in the autocorrelation, also standard deviation (STD, 2nd row plot in Fig. 1) can increase before a critical transition, caused by the systems tendency to move further away from its stable state when losing stability. The proximity to an alternative equilibrium and the resulting attraction can also cause asymmetries in variance and the occurrence of short jumps to states further away from the stable state and back, so-called flickering. Such effects can be identified by changes in skewness and kurtosis (3rd row plot in Fig. 1). Serial correlations between successive time samples can also induce changes in spectral density, so called reddening, measured by spectral analysis (FR, 7th plots in Fig. A4 and A5). And finally, correlations over longer time-scales than detectable with AR1 can be measured by de-trended fluctuation analysis (DFA, bottom plots in Fig. A4 and A5) These and some more signs are comprised under the term Early Warning Signals (EWS) [33].

However, in practice not all systems prone to CTs show easily discernible EWS [2], [17] Often, the signals remain hidden behind stochastic [3], [4] or topological [18] particularities or they are so weak that predictions easily err.

Several issues in this regard have been reported. They range from the noise-dependency of EWS, causing clear indications of tipping with a low noise-factor and blurred signals when the noise is increased [19], via the so-called hysteresis of complex systems making shifts occur at different values of slowly drifting parameters in dependence on whether the tipping is approached from the one or the other equilibrium [17], up to the problem of interpreting EWS unambiguously in larger distances to the CT. A particularly grave problem for the detection of EWS is additionally caused by the fact that many systems not being prone to CTs may show EWS-similar signals in their own right [17]. As it could be shown that removing the trend from the time series can prevent such false positives, the data in this investigation has been consistently detrended before analysis (see Fig. 1, and for details next section).

Together, these difficulties in the interpretability of EWS provide reason to consider possibilities for making the prediction of imminent regime shifts more reliable. With this aim, we tested the possibilities of predicting CTs with the help of automated decision tools, in particular with artificial neural networks (ANNs). A particular interest thereby concerned the possibility to compare the effects of causal CT-triggers on the micro-level of interaction to the aggregated statistical signals of the overall system states. For this reason, and deviating from the bulge of existent EWS-investigations, we deployed agent-based methods to simulate the system in question, in this case a Repeated Public Good Game constellation, which is driven by feedbacks from agents’ individual learning and social contagion, the details of which are explained in the next section.

Deploying an ABM for a fine-grained access to the system's micro-level interactions provides one decisive advantage for the generation of data. Training ANNs for classification tasks needs positive as well as negative instances of an interesting system behaviour. In the case of tipping (i.e. systems undergoing a CT) it is not always easy to differentiate these instances beforehand, since signals of imminent regime shifts can distribute over a wide range of system states. When considering a slowly drifting parameter on aggregate level, as usually done in EBMs, it is thus difficult to specify when the system is still predominantly under the influence of an equilibrium – and thus not expected to show EWS – and when it is already prone to tipping and EWS can be expected. Different to this, in ABMs the very dynamics that cause the shift are considered on the micro-level of component interaction and thus can simply be paused from driving the system towards a tipping. In this case, the component interactions still generate data, but the behaviour shifting dynamics are missing. The time series obtained in this way simply represent steady state noise of the system at rest, thus providing negative instances in arbitrary length with similar characteristics as the ones considered as positives for training the classifier. The details of this time series generation and of the deployed alternative will be explained in the next section.

## The model used for generating data

In preparation of the investigation, an agent-based model (ABM) was used for generating two training sets of time series, the one consisting of about 60.000 and the other of 80.000 instances, both taken in varying distances to CTs. The ABM simulates a stylized form of a Repeated Public Good Game (RPGG), in which agents adjust an individual probability for contributing a.) according to their payoffs in relation to payoffs from preceding rounds of the game and b.) according to the majority of other agents’ respective behaviour.

An RPGG is a well-known game theoretic formalization of a situation in which cooperation can procure a common good of high social value, but contribution is impeded by the possibility to realize still higher payoffs by free riding [8]. Due to social contagion, this situation when repeated tends to evolve towards a socially sub-optimal Nash-equilibrium of pervasive defection [27] with nobody being willing to cooperate as long as nobody else does [9], [22]. Although experiments show that contributions in RPGG do not decline to absolute zero [9], [10], [23], changes in cooperation behaviour can be quite abrupt [11], in particular when feedback-driven reciprocal entrainment is involved [35].

Our model mimics these feedback-driven shifts in a simple way, which is primarily tuned for generating CT time series, and not for modelling realistic behaviour. A population of *N* agents are set up, each agent playing the RPGG with all others. The individual cooperativeness depends on their contribution probability *c_i_*, with a high initial cooperativeness being normally distributed close to 1 ($c_{i}=1-s$with *s* ∈ {0.02, 0.04}). While running the model, agents in each iteration, dependent on *c_i_*, invest either all or nothing of an endowment into a common pool, which after investment of all is multiplied with an enhancement factor *f* and is evenly distributed among all agents earning them a final payoff *π_i_* dependent on investment. As usually [28], the payoff of an agent *i* is defined as

(1) $$\pi_{i}=E-I_{i}+f\frac{\sum_{j=1}^{n}I_{j}}{N},$$

with the initial endowment *E*, the individual investment *I_i_*, the number of participants *N* in the game, and an enhancement factor *f*, expressing the added value from social cooperation.

Playing the RPGG in successive rounds, the dynamics of the contribution probability are given by a simple influence mechanism according to which agents adapt their contribution probability. Agents compare their current payoff with the one of the preceding round of the game. If the payoff decreases, they adjust their contribution probability in respect to the fraction of other agents who did not contribute to the public good. The contribution probability of each agent is updated according to:

(2) $$\begin{array}{ccc} & & \text{if}\;\pi_{i,t-1}>\pi_{i,t}\;\;\text{then}:\;c_{i,t+1}=c_{i,t}-g{\left(\frac{{N|}_{I=0}}{N}\right)}^{3},\\ & & \text{else}:\;c_{i,t+1}=c_{i,t}, \end{array}$$

with the number of non-contributors (free riders) in the population ${N|}_{I=0}$, and an arbitrary scaling factor *g* which governs the dynamics of the development. The dynamics generate a distinct downwards-tipping of the number of cooperators after a long and slow decline (see top plot in Fig. 1).

In order to account for the possibility of an upwards-tipping (i.e. a backward change from the Nash-equilibrium to the social optimum of near overall cooperation), which was found to enhance the generalizability of ANN-training (and apart from this is a common feature in EBM-simulated systems used for EWS-analysis, see a.o. [19]), we assumed a near-symmetric reinvigoration dynamic for cooperation. Once the majority cooperation is lost and the RPGG is close to its Nash equilibrium, the difference between the actual payoff and the payoff the agents could receive with a majority investing might cause individuals to be dissatisfied, leading to a reinvigoration of cooperation. In this case, the dynamics of (2) are suspended and the update of the contribution probability is given by:

(3) $$\begin{array}{ccc} & & \text{if}\;\pi_{i,t-1}>\pi_{i,t}\;\text{then}:\;c_{i,t+1}=c_{i,t}+g{\left(\frac{{N|}_{I=1}}{N}\right)}^{3},\\ & & \text{else}:\;c_{i,t+1}=c_{i,t} \end{array}$$

Together with the downward tipping this generates time series expressing the development of the fraction of RPGG-contributors (cooperators) in the overall population, as the one depicted in the top plot in Fig. 1.

The RPGG-ABM was implemented in Netlogo (https://ccl.northwestern.edu/netlogo/) and actuated with the Python modul pyNetlogo (https://pynetlogo.readthedocs.io/en/latest/) for generating two sets of training data. For the first set, 500-step-series were extracted every 10 steps in distances ranging backwards from 100 to 700 steps before the tipping point (the CT) from an overall set of about 1000 series with downwards and upwards-tippings as shown in the top plot in Fig. 1. To generate these, the simulations were varied in respect to an initial deviation of *c_i_* from 1 ($c_{i}=1-s$with *s* ∈ {0.02, 0.04}) and in respect to the size of the simulated population randomly varied in an interval between 200 and 500. From the roughly 120.000 500-step chunks, a random sample of 30.000 was selected for being used as positive instances for training the classifier. Additionally, another 30.000 such 500-step chunks were generated by rendering Eqs. (2) and (3) ineffective. Without influence on the agents’ cooperation probability, the dynamics remained steady while showing similar statistical properties as the tipping ones. These steady state time series chunks were used as negative instances in training the classifier.

The second set of training data was generated for comparison. In this case, 500-step chunks were extracted every 10 steps from the whole range of the generated series and checked for their auto-correlation at-lag-1 (AR1). Since usually, time series are considered correlated when AR1 exceeds a value of 0.3, we took those 500-step chunks with an AR1 > 0.4 as positive instances (i.e. indicating an imminent CT) and those with AR1 < 0.1 as negative instances (i.e. not indicating an imminent CT). From these collections again, random samples of 40.000 each were taken for training the neural network, as detailed in the next section.

In order to avoid false positives of EWS (see Section 2) and having the ANN learn superficial features, like for instance the overall trend of the time series, all considered time series were detrended with a Savitzky-Golay filter [30] with a window length of 101 and a polynomial order of 5 (see Fig. 1, for comparison, EWS-analyses of an example time series in un-detrended and detrended form is shown). The detrending method with these settings was chosen after extensive tests with different filters and different parameters, showing that polynomial order and window size have no influence on the results of EWS-analysis in this case. (Examples of these tests are shown in the appendix, including also results for the coefficient of variation, for spectral analysis and for detrended fluctuation analysis). Additionally, since upward as well as downward tipping time series were considered, time series were scaled using scikit-learn's Standard scaler (sklearn.preprocessing.StandardScaler.html) before being applied to the decision tool.

## The deployed methods

For assessing the onset of tipping in RPGG time series with automated decision tools, several possibilities were considered. Preliminarily tests were made in respect to the generalizability of classifications with data from very different systems. For this we deployed solely machine learning tools as contained in Python's *scikit-learn* toolkit. In another experiment, following a suggestion by [39], time series were transformed into so-called recurrence plots and exposed to Convolutional Neural Networks for classification. In parallel, time series were also screened with the Python package *tsfresh* (https://tsfresh.readthedocs.io/en/latest/#) for automatically calculating a large number of time series characteristics, which allows evaluating their explanative power and importance in classification tasks. These experiments however, did not yield clear results so far. They will be presented elsewhere.

In the classification experiment at hand, a so-called Long-Short-Term-Memory (LSTM) neural network was deployed. Originally proposed to avoid the vanishing or exploding gradient problem [15], these ANNs are considered efficient learners in the context of long-term patterns in time series [12]. Their architecture foresees particular components, “cells” and “gates”, which regulate the flow of information from neuron layer to neuron layer with the effect of enabling the ANN to consider a sort of context knowledge when being trained on new data, thus accounting for a sort of experience from earlier phases in their training and making them particularly sensible for subtle changes in developments.

The LSTM was implemented using *keras* (version 2.2.4, keras.io) based on *tensorflow* (version 1.13.1, tensorflow.org), consisting of a dense input layer with 500 neurons and Relu-activation, an LSTM-layer with 100 neurons, another dense layer with sigmoid-activation for the output (thus with just one neuron) and a dropout-rate of 0.2. In order to fit the model to data an Adam-optimizer was used, which yielded a test-set-accuracy (test-data size = 20%) of 98.7% when applied to the first dataset and 88.9% when applied to the second one.

Our intuition was to explain the high accuracy on the first data set with the fact that positive and negative instances used for training show rather clear distinctive features, with the positive ones being taken from truly tipping dynamics and the negative ones representing just the steady variance of initial cooperation probabilities. This was the reason to generate another data set for comparison with the results of a separate training.

## Results on unseen data

As it turned out, in both cases the classifiers proved quite efficient on completely new data as well.

For testing their predictive power separately from the data used for training, several other sets of time series were generated with the RPGG-model varied in respect to population size and the deviation from initial cooperation *s*. All of them were detrended and scaled in the same way as the training data, but this time, time series of greater length were applied to the trained LSTMs. Predictions were taken over a minimum of 1800 and a maximum of 3500 (in the networked cases) time steps in rolling windows of size 500 every 10 time steps up to the tipping point (with the initial 500 steps omitted for considering equal window sizes).

**Table 1:** LSTM-classification results of test data Dataset 1, negatives generated by rendering Eqs. (2) and (3) ineffective: 60.000 instances. Dataset 2, generated by differentiating according to AR-1 > 0.4 and AR-1 < 0.1: 80.000 instances.

**Fig. 2:**
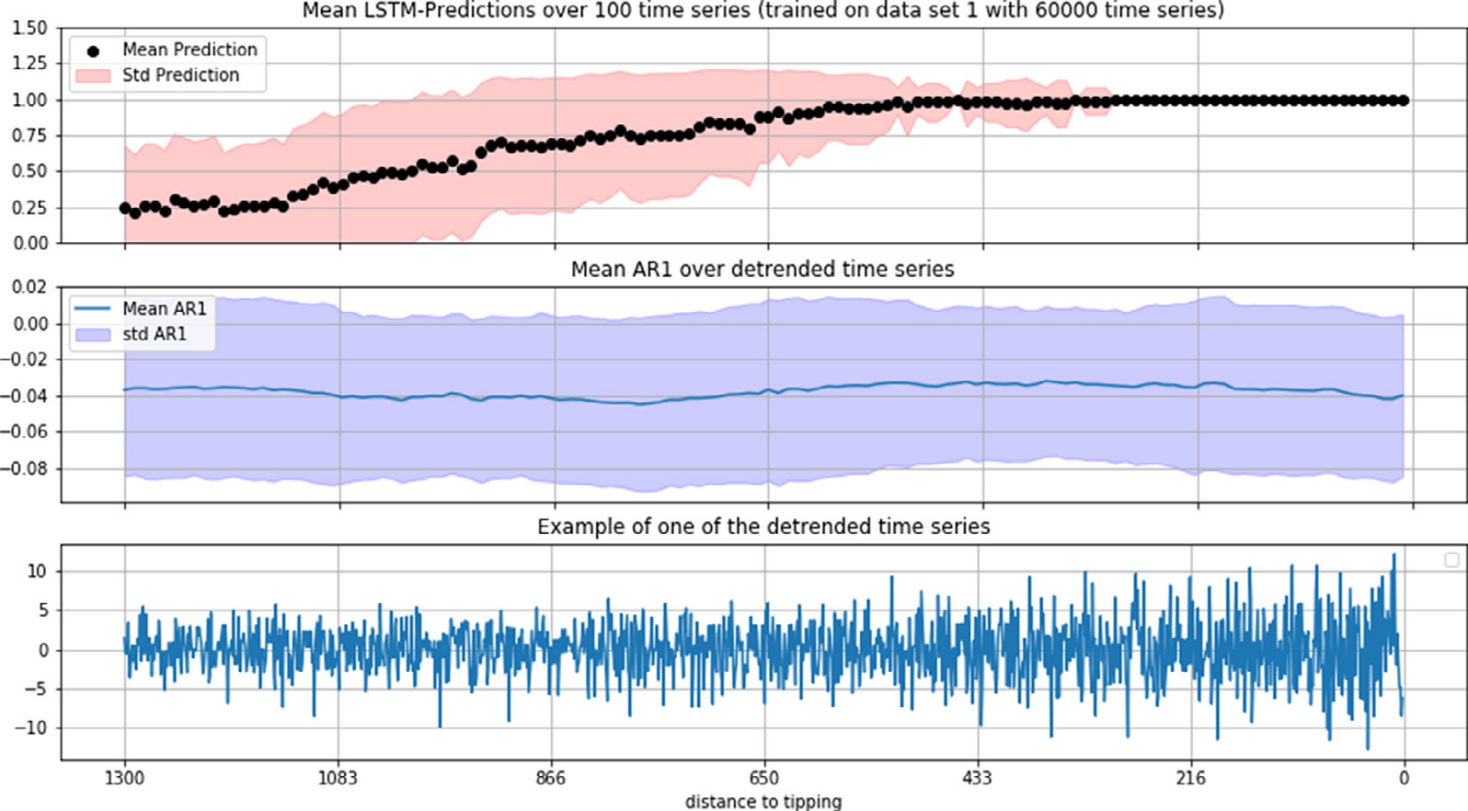
Top-plot: mean LSTM predictions over 100 detrended time series of length 1300 of a grid-based version of the RPGG. Middle-plot: mean auto-correlation at lag-1 of the detrended time series (showing no indication of an imminent regime shift). Bottom-plot: residuals of one example time series,.

An example result for the predictions of an LSTM trained on the first data set on a previously unseen time series from a grid-based version of the RPGG is shown in the bottom plot of Fig. 1. The top plot in Fig. 2 shows an average of such predictions over 100 time series, which clearly runs up to 1 when the system approaches the tipping point (from left to right in the plot), indicating the LSTM's positive classification of an imminent regime shift well before the residuals of the example time series, shown in the bottom plot in Fig. 2, show optically detectable signs of changing dynamics. At the same time, the mean auto-correlation at-lag-1 (AR1, Fig. 2 middle plot) of these detrended time series (that is, the indicator usually most commonly referred to in EWS-analysis) does not show any indication of an imminent regime shift at all, similar to most other EWS-indicators we tested (that is, standard deviation, skewness, kurtosis, coefficient of variation, reddening and detrended fluctuation. See the fifth and sixth row plots in Fig. 1 and the additional analysis in Fig. A4,A5 and A6 in the appendix). The performance of the LSTM trained on the second data set is shown in Fig. A4 in the appendix, together with results for the other tested EWS-indicators. In our tests, none of them apart from standard deviation and its relative variant, the coefficient of variation (see A6 in the appendix), were showing signs of EWS. Standard deviations by themselves however, often are not considered unambiguous signals. Most often in literature, at least two independently changing EWS-indicators are requested as clear indication for the approach of a tipping, with standard deviation being most commonly considered together with auto-correlation at lag 1.

### The networked case

As mentioned, a particular interest of these investigations concerned the possibility of considering details of micro-level interactions with ABM-simulations, which are not visible on the (usually considered) level of aggregated system dynamics as assessable with EBM-methods. With this interest, we implemented the RPGG-ABM, as described in Section 3, in different network settings, with the agents, differently to the grid version, receiving their cooperation-determining social influences not from the fraction of free riders (respectively cooperators) of the whole population, but just from the fractions among their link-neighbours. The idea behind this was to see, if the classifier as trained on the grid-version is able to distinguish the influence of different subsets of the network on the dynamics of cooperation.

**Fig. 3:**
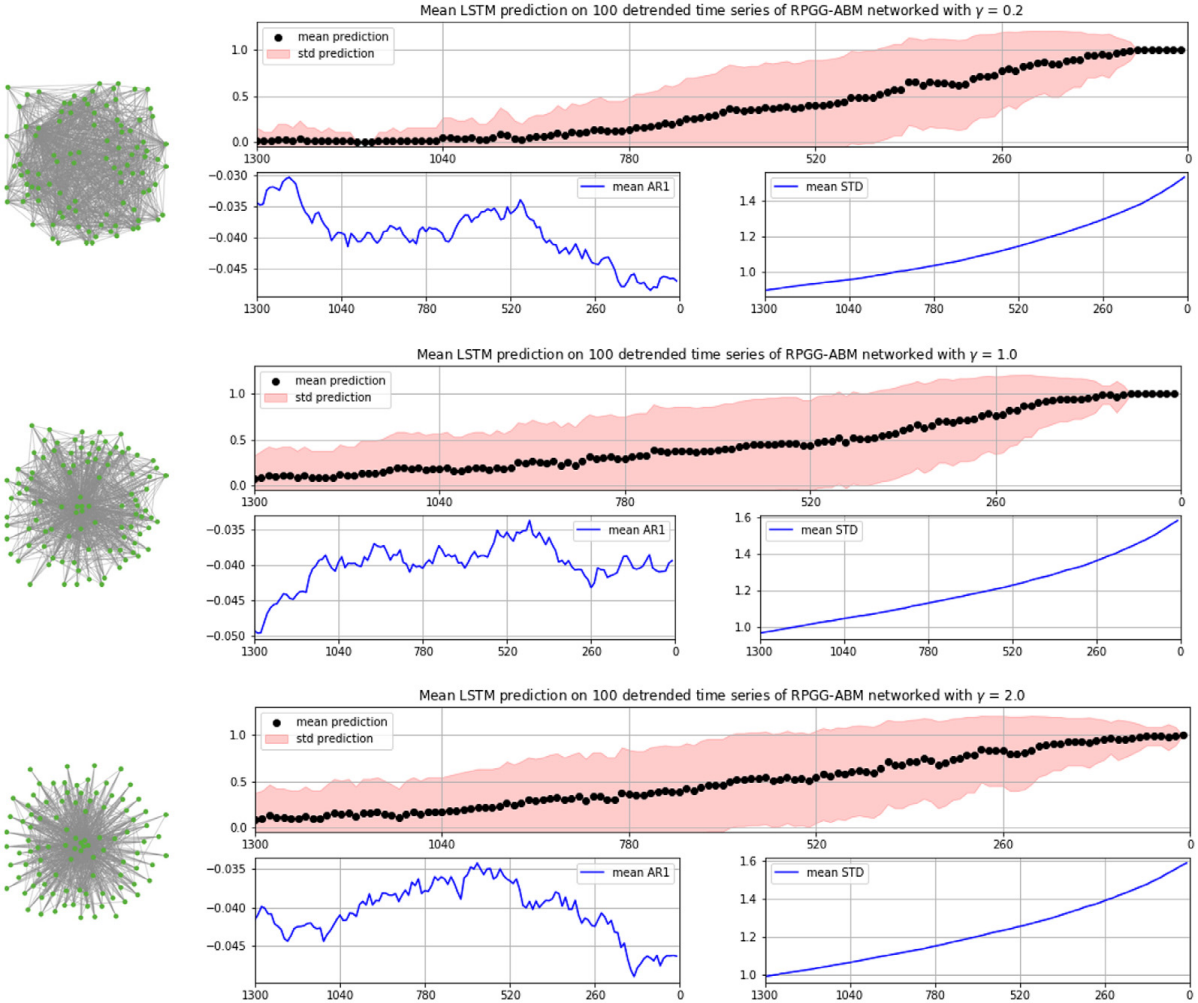
Left: Realizations of scale-free networked representations of ABM-RPGG-populations, in topologies as described in Goh, Kahng, and Kim [14] with (from top to bottom) γ=0.2, γ=1 and γ=2. Right: Corresponding LSTM predictions of time series generated from networked versions of the RPGG's total population, with AR1 and STD shown below, averaged over 100 instances each. As in Figs. 1 and 2, while STD is increasing, AR1 does not show any indication of an imminent CT.

This setting was realized in a scale-free network implemented after the suggestion of Goh, Kahng, and Kim [14], which, dependent on a parameter *γ* varying between 0 and 2 generates networks with a distribution of link degrees obeying a power law $P\left(k\right)∼k^{-\gamma }$ (see also [18]). The left images in Fig. 3 show realizations of such networks with γ=0.2, γ=1 and γ=2. With these different *γ*-values, another set of time series from fractions of cooperators was generated, varied as before in the size of the population, the initial deviation s from the cooperation probability $c_{i}=1$ (see Section 3) and in whether they were tipping from upper to lower equilibrium or vice versa. Fig. 3 shows the mean results for, in each case, 100 of these time series, which were applied to the LSTM trained on the second data set in this case (results again being very similar to the ones of the first data set).

As can be seen, the certainty about an imminent regime shift runs up to 1 significantly later than in the grid-based version in this case. However, and somehow contra-intuitively, the dynamics of the network with the most skewed link distribution (γ=2) seem to be slightly earlier classified as approaching a CT than the ones with γ=0.2. As in the tests on the grid-based version, the mean auto-correlation of the detrended time series does not show any signs of an imminent CT, while mean STD is clearly increasing.

**Fig. 4:**
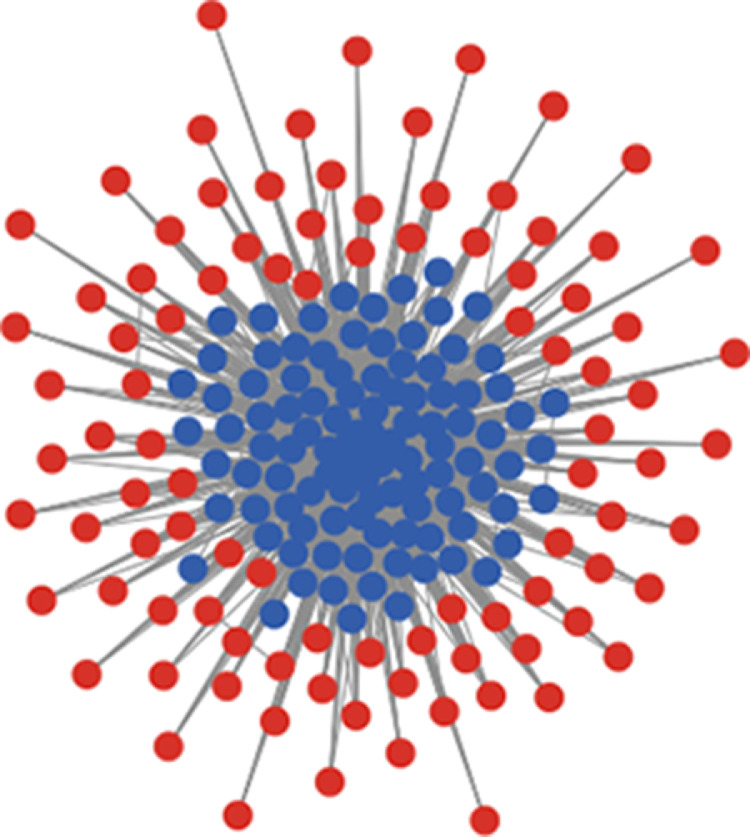
Instance of Goh-scale-free-network with γ=2, with nodes with above-median Eigenvector-centrality in blue and nodes below-median in red.(For interpretation of the references to color in this figure legend, the reader is referred to the web version of this article.)

In order to dig into more detailed levels of agents’ interactions, we analysed the social networks of the RPGG in regard to the centrality of agents and distinguished the development of the fraction of cooperators with above-median centrality from the one of the fraction with below median-centrality. In these tests Closeness- and Eigenvector-centrality were considered, with the first indicating the property of being “close” in terms of shortest paths to other nodes in a network, and the second indicating the influence of a node in a network based on the number of connections this node has to other highly influential nodes in the network [29]. In order to illustrate this differentiation, Fig. 4 shows an instance of a Goh-scale-free-network with γ=2, with nodes with above-median Eigenvector-centrality in blue and nodes below-median in red.

**Fig. 5:**
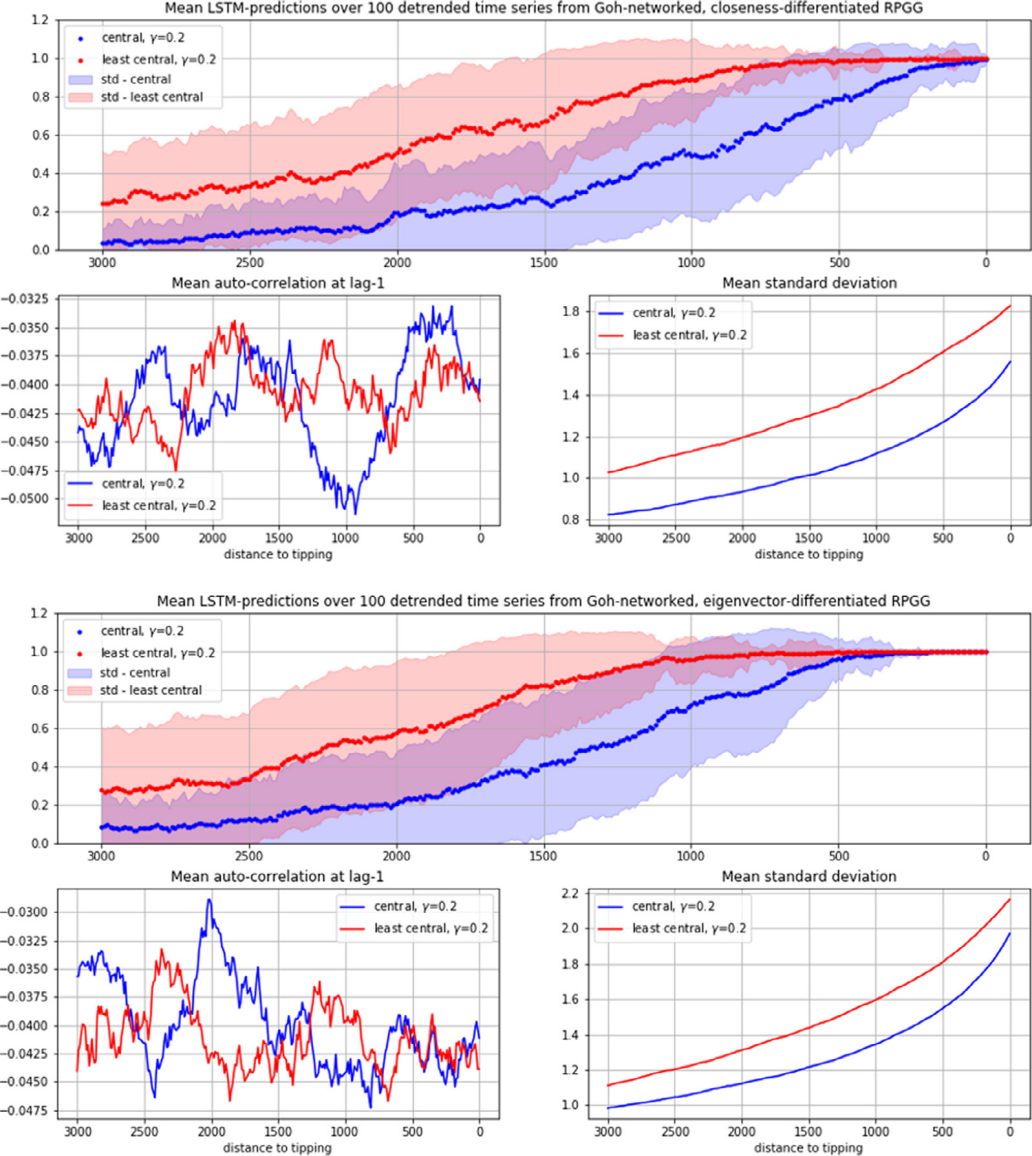

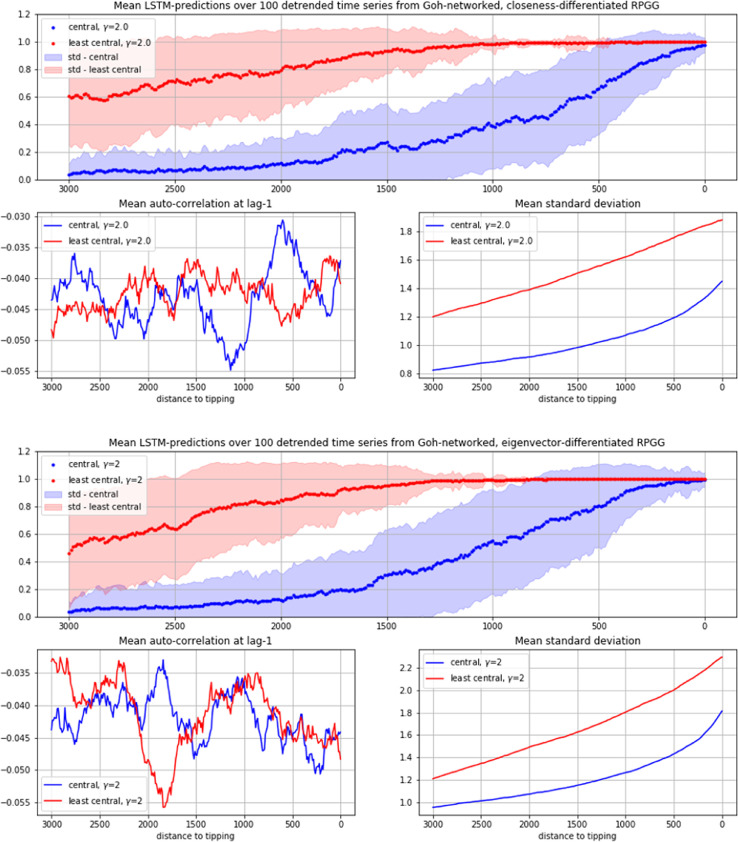
Mean LSTM-predictions over 100 time series, each of above-median (blue) and below-median centrality (red) fractions of Goh-networked RPGG-cooperators (standard deviation in light-blue and light-red), together with mean AR1 and mean STD (smaller plots). Top and second row from Goh-networked RPGGs with γ=0.2, third and bottom row with γ=2. Top and third row differentiated by Closeness-centrality, second and bottom row by Eigenvector-centrality.(For interpretation of the references to color in this figure legend, the reader is referred to the web version of this article.)

**Fig. A1:**
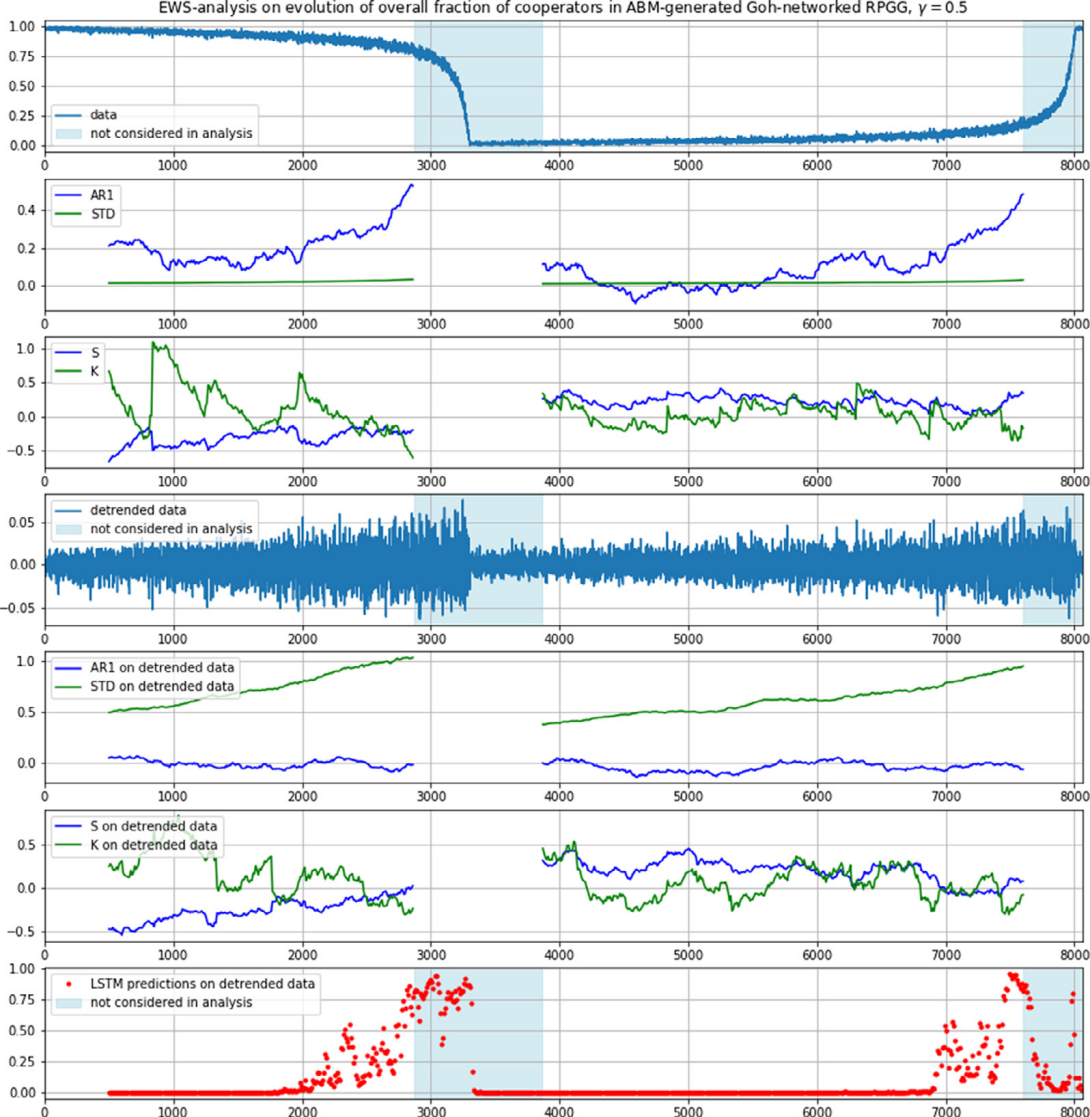
Top plot: example of one of the generated time series showing loss and reinvigoration of cooperation in the total population of a Goh-networked version of the RPGG (% of co-operators in the population). Second row plot: AR1 and STD of the un-detrended time series shown in the top plot, with data immediately before the tipping and shortly afterwards (light blue) not considered to avoid rolling window miscalculations. Third row plot: S and K of the un-detrended time series. Fourth row plot: detrended time series of top plot, using a Savitzky-Golay filter with window length 101 and a polynomial order of 5. Fifth and sixth row plots: AR1, STD, S and K of detrended data. Bottom row plot: LSTM prediction about imminent CT on the detrended time series in the fourth row plot.(For interpretation of the references to color in this figure legend, the reader is referred to the web version of this article.)

**Fig. A2:**
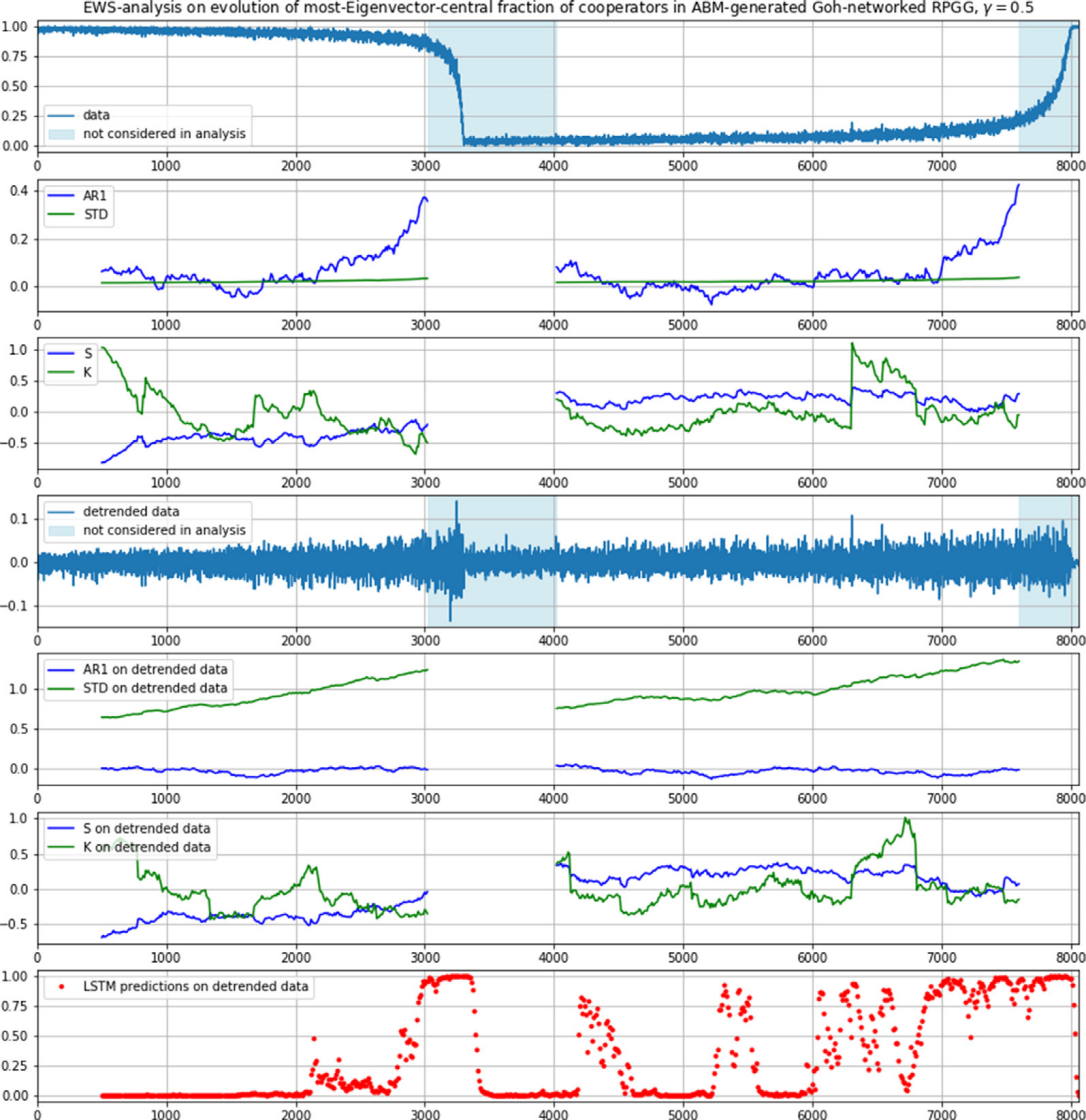
Loss and reinvigoration of cooperation in the **most** Eigenvector-central 50%-fraction of the population of a Goh-networked version of the RPGG. Plots in analogy to Fig. A1.

**Fig. A3:**
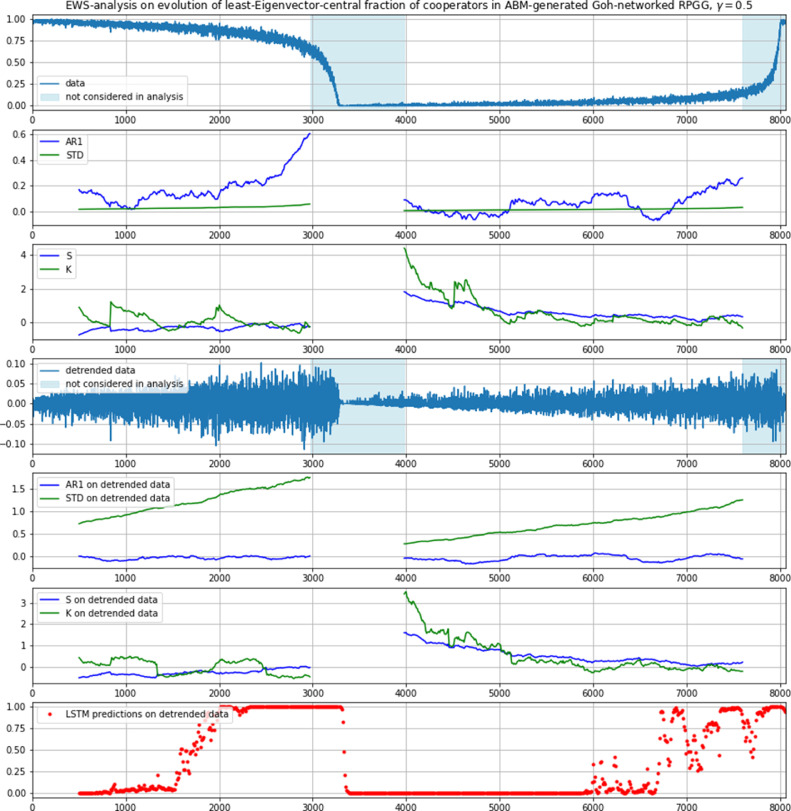
Loss and reinvigoration of cooperation in the **least** Eigenvector-central 50%-fraction of the population of a Goh-networked version of the RPGG. Plots in analogy to Fig. A1.

**Fig. A4:**
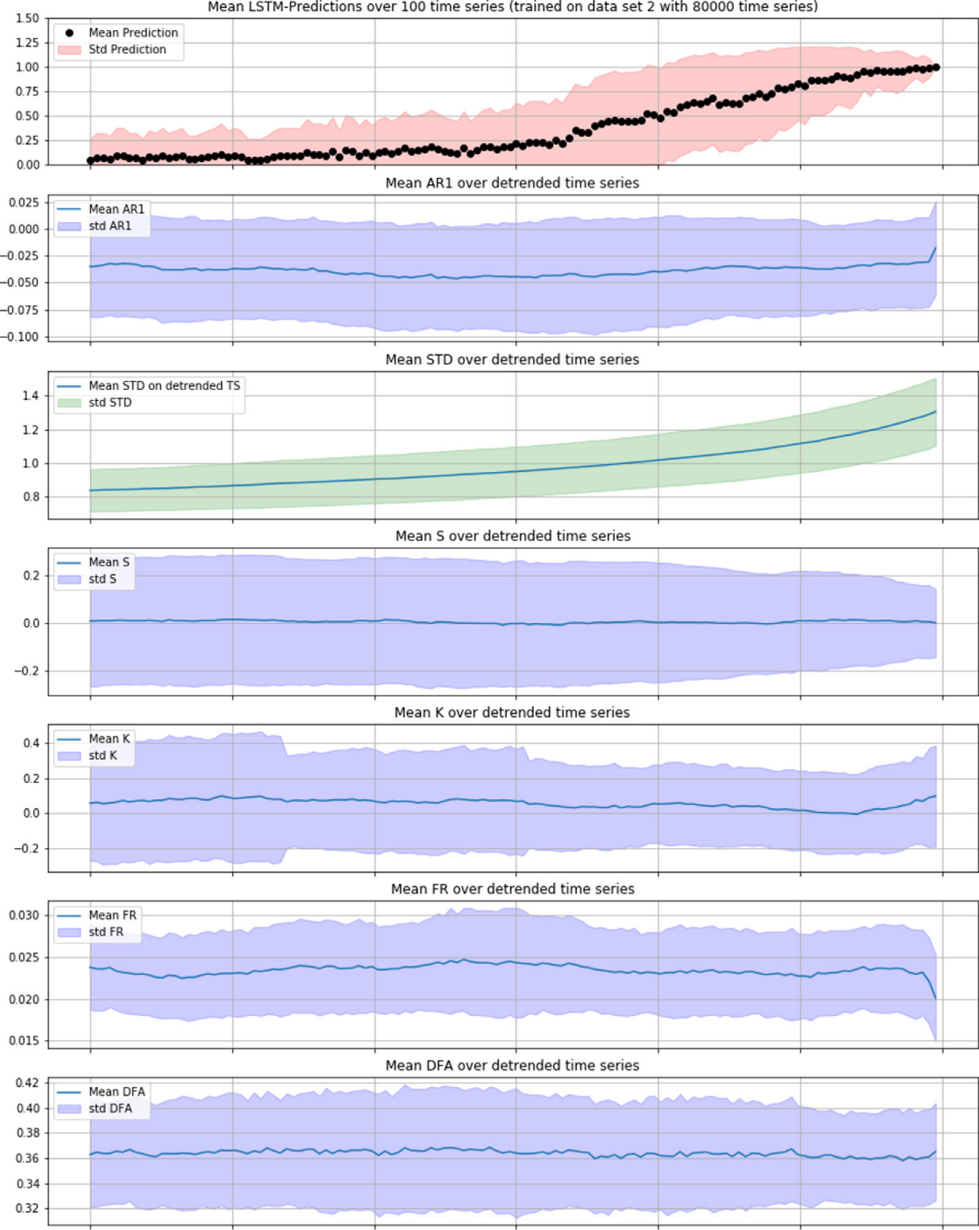
Top: Mean LSTM predictions, averaged over 100 time series (similar to the ones shown in Fig. 1), with the LSTM trained on data set 2 with 80,000 instances (as described in Section 3). Below: mean results of EWS analysis: auto-correlation at lag-1 (AR1), standard deviation (STD), skewness (S), kurtosis (K), reddening (FR), detrended fluctuation analysis (DFA)of the same 100 time series.

**Fig. A5:**
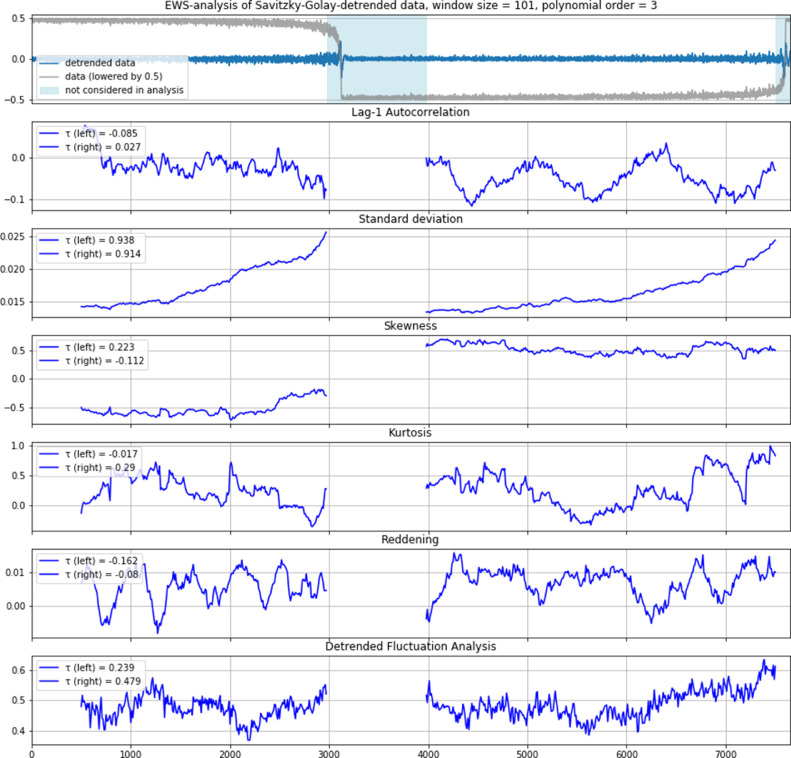
Example analysis of alternative Savitzky-Golay-generated residuals (blue in the top plot), here with a polynomial of order 3, from an ABM-generated time series showing loss and reinvigoration of cooperation in an RPGG. The plots indicate the Mann-Kendall-trend τ [13] of the indicators, conveying an idea of whether the curves show a monotonic upward or downward trend and thus could be seen as showing EWS.

**Fig. A6:**
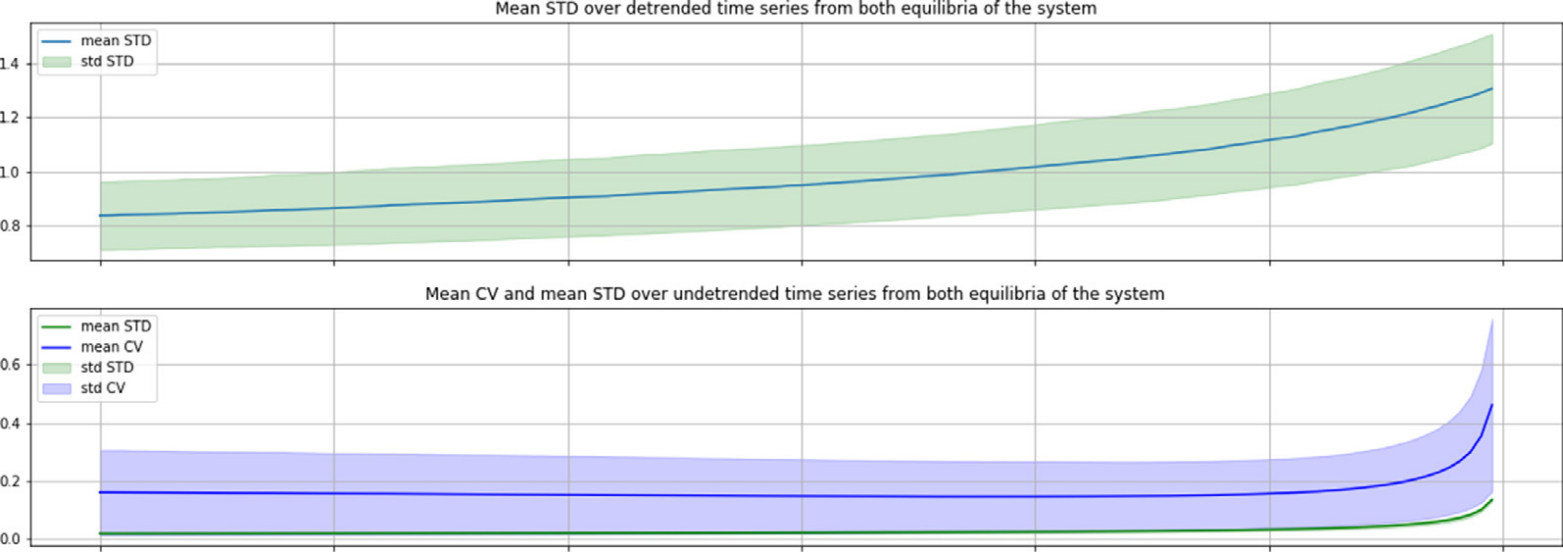
Comparison of mean coefficient of variance (CV) over un-detrended data with mean STD over detrended and mean STD over un-detrended time series from both equilibria of the system. Note that CV (aka relative STD), defined as ratio of standard deviation to mean, does not suggest detrending, but is sensitive to the local mean of the data. Therefore, applying CV to the complete test set of 100 un-detrended time series from both equilibria of the system shows a striking difference to mean STD on the same un-detrended data (as can be exemplarily seen in the bottom plot in A6 for data set 1). Note however, that this data set contains time series from the lower and from the upper equilibrium of the tested system, and hence two sets of time series with different means. When this set is separated and only time series from one of the equilibria are considered, the difference between STD and CV becomes much smaller and less indicative (as can be exemplarily seen in the bottom plot of A7 for time series from the lower equilibrium of data set 1). For this reason, the main part of the paper omits CV-analysis and focuses predominantly on detrended data.

When exposed to LSTM-prediction, the time series from both networks yield very similar results (see Fig. 5). As in the case of the total population (Fig. 3), the LSTM's estimation of seeing EWS increases clearly when approaching the tipping point (from left to right). What is interesting in the centrality-differentiated case however, is that more information about the imminent regime shift seems to be mediated by the below median fraction of the population (see also Figs. A1, Fig. A2, Fig. A3 in the appendix). As can be seen, as well in the γ=0.2 as in the γ=2 case the certainty of predictions for the below median fraction rises significantly earlier than the one for the above median fraction, with the γ=2 case (that is, the highly centralized network case) showing particularly striking differences. If these results prove generalizable, they may suggest focusing attempts of predicting forthcoming regime changes in cooperation networks on the peripheral nodes of the network.

Note that, as mentioned in Section 3, all investigated time series are analysed in a (Savitzly-Golay) detrended form. While in the often successfully used combination of EWS-indicators AR1 and STD, only STD shows an increase that could be seen as an indications for an approaching regime shift (as well in the fraction of highly central as in the low-central nodes), the LSTM appears to have learned to recognize respective differences nevertheless, although it was trained in this case only on time series distinguished along the AR1 > 0.4 (positives) and AR1 < 0.1 (negatives) difference. These results suggest that using automated decision tools for detecting imminent regime shifts in complex systems with more than one equilibrium state may efficiently complement and support EWS-methods as they are considered so far.

## Summary

We report on attempts of training an LSTM-ANN for predicting critical transitions in time series generated with an ABM-model of a Repeated Public Good Game. Different from the bulk of existent EWS-investigations on simulated systems with alternative equilibrium states, the focus in this case is on the possibility to assess indications of arising regime shifts on the micro-level of agent interactions. For this, the trained LSTM was applied to different time series from scale-free networked versions of the RPGG, among them time series taken from two different fractions of the population differentiated by above and below median-centrality of their social networks. The investigation showed that the trained LSTM is able to identify signals of approaching critical transitions in detrended time series, to which the usually deployed EWS-indicators do not always respond unambiguously. What is more, we found indications that the LSTM's estimation for seeing such signals increases earlier with regard to the low-centrality fraction of a population, which suggests to focus further investigations of regime-shift-predictions in social systems on the periphery of these systems.

**Supplementary material *and/or* Additional information:** *[OPTIONAL. We also give you the option to submit both supplementary material and additional information. Supplementary material relates directly to the work that you have submitted and can include extensive excel tables, raw data etc. We would also encourage you to include failed methods or describe adjustments to your methods that did not work. Additional information can include anything else that is not directly related to your method,* e.g. *more general background information, useful links etc. Introduction is not a section included in the MethodsX format. This information could be moved to the end under Additional Information.*

## Figures and Tables

**Fig. A7:**
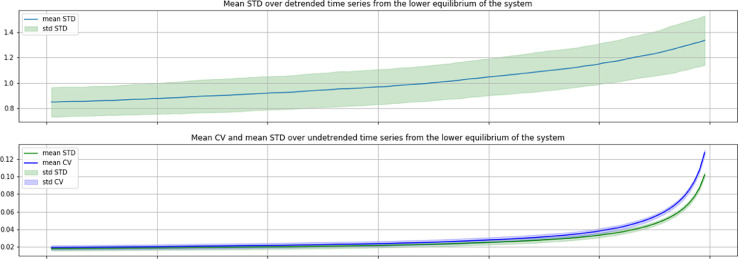
Comparisons of mean CV over undetrended data with mean STD over detrended and mean STD over un-detrended data for time series from the lower equilibrium of data set 1, as described in section 3.
